# Editorial: Soil biota and climate smart crops

**DOI:** 10.3389/fpls.2023.1250831

**Published:** 2023-07-19

**Authors:** Acga Cheng, Gordana Gajic, Febri Doni

**Affiliations:** ^1^ Institute of Biological Sciences, Faculty of Science, Universiti Malaya, Kuala Lumpur, Malaysia; ^2^ Department of Ecology, Institute for Biological Research `Sinisa Stankovic`, National Institute of Republic of Serbia, University of Belgrade, Belgrade, Serbia; ^3^ Department of Biology, Faculty of Mathematics and Natural Sciences, Universitas Padjadjaran, West Java, Indonesia

**Keywords:** bacterial diversity, crops, gene profiling, mycorrhiza, nutrient acquisition, root-microbe interaction, soil properties, transcriptomics

Soil biota consists of an incredible diversity of organisms, including microorganisms, soil animals, and plants that live entirely or partially in or on the soil or pedosphere (Harman et al., 2021). Despite the fact that there are millions of soil organism species, only a small fraction of them have been cultured and identified (Ge et al., 2023). Hence, the goal of this Research Topic is to create a collection of works that highlight recent advances in soil biota research and crop development, particularly for crops that have the potential to be employed as climate smart crops. This Research Topic contains four Original Research articles, each of which offered new insights on how soils or plants will fare in a rapidly changing climate.

In recent years, there has been an increased research focus on the influence of soil organisms on crop health in order to dissect the relationships between biota diversity and key crop phenotypic traits, such as disease resistance and growth promotion, under a rapidly changing climate (Abdullah et al., 2021). However, reports on the interactions between soil biota and plants in assisting the development of crop management strategies are generally still lacking. The study conducted by Kumar et al. attempted to identify keystone taxa and core microbiota in in the wheat (*Triticum aestivum*) rhizosphere, as well as their response to soil physicochemical properties, allowing the exploration of approaches for manipulating core microbiota for improved plant growth and yield. This is the first study of its kind in India which includes wheat, one of the world’s big three cereals, along with rice (*Oryza sativa*) and maize (*Zea mays*). Their research revealed the existence of bacteria that are beneficial to plants (such as *Flavobacterium* and *Flavisolibacter*) in the wheat rhizosphere beneath the Indo-Gangetic plain. Additionally, their findings shed light on how pH and readily available nutrients affect the diversity of bacteria in the wheat rhizosphere.

It has been demonstrated that soil biota, the primary determinant of resource efficiency in agriculture, can increase agricultural sustainability in various ways, including by increasing crop output and nutrient uptake while reducing nitrogen leaching losses (Shivay et al., 2022). Understanding how these components can aid in the optimization of soil biota-plant interactions is crucial for crop improvement in a changing climate. In order to further understand the mechanisms underlying root-microbe interactions governing crop phosphorus acquisition, Zhang et al. investigated root morphological and exudation traits of four crops, including bok choy (*Brassica chinensis*), tomato (*Solanum lycopersicum*), lettuce (*Lactuca sativa*), and cowpea (*Vigna unguiculate*), in response to microbial phosphorus in a pot experiment with various straw and phosphorus additions. They reported that the high abundance of microbes was related to the short roots of the four studied crops. While their study demonstrated that straw addition stimulated the growth of bacteria and fungi to increase microbial phosphorus, root phosphorus-acquisition strategies in response to microbial phosphorus dynamics differed among the four crops evaluated following straw addition. It is important to note that to ensure strong crop growth, high-phosphorus fertilisation may need to be considered in addition to returning straw.

When developing crop management strategies based on improving soil biota-plant interactions, it is critical to consider the farming system and crop association (Masson et al., 2022). Zhou et al. investigated how soil organic carbon changes in perennial mugwort and the underlying mechanisms during different cropping years. Their findings indicate that short-term rather than long-term perennial mugwort cropping could benefit soil carbon sequestration and help China achieve its carbon neutrality target. Perennial crops typically have an enormous potential to trap more carbon into agricultural soils and hence ameliorate climate change, and they account for 30% of global croplands with higher biomass than conventional crops.

Plants are known to have diverse strategies for acquiring mineral nutrients (or foraging), such as mycorrhizae and root system architecture modification (Herms et al., 2022; Pantigoso et al., 2022). Despite substantial research on individual strategies in laboratory or greenhouse settings, little is known about how plants coordinate these strategies in the field. Utilizing a cross-ecosystem transcriptomics approach on maize, Sugimura et al. unveiled novel insights into understanding gene-environment interactions in plant foraging strategies in complex environments by defining the three gene co-expression modules for root development, mycorrhiza formation, and phosphate starvation response. According to their findings, the consistency of the relative expression levels of module member genes across genotype site combinations imply that the genetic modules described by this approach are strongly and robustly regulated across ecosystems and that their regulatory systems are species-specific.

The articles included in this Research Topic, individually and collectively, contribute significantly to our knowledge of soil biota and their role in combating climate change and improving agricultural sustainability. There is a need to recognise that the application of modern biotechnology technologies, such as genome editing, can be even more effective when assisted by effective microbes, which is a field ripe for future research (Loo et al., 2022).

## Author contributions

This Research Topic was proposed by AC. Each submission was subject to assessment by the editorial board as well as peer reviewers, and all of the editors collaborated to decide which articles were accepted. AC oversaw this editorial introduction. GG and FD offered thoughts and edits to help shape the published document. All authors contributed to the article and approved the submitted version.
